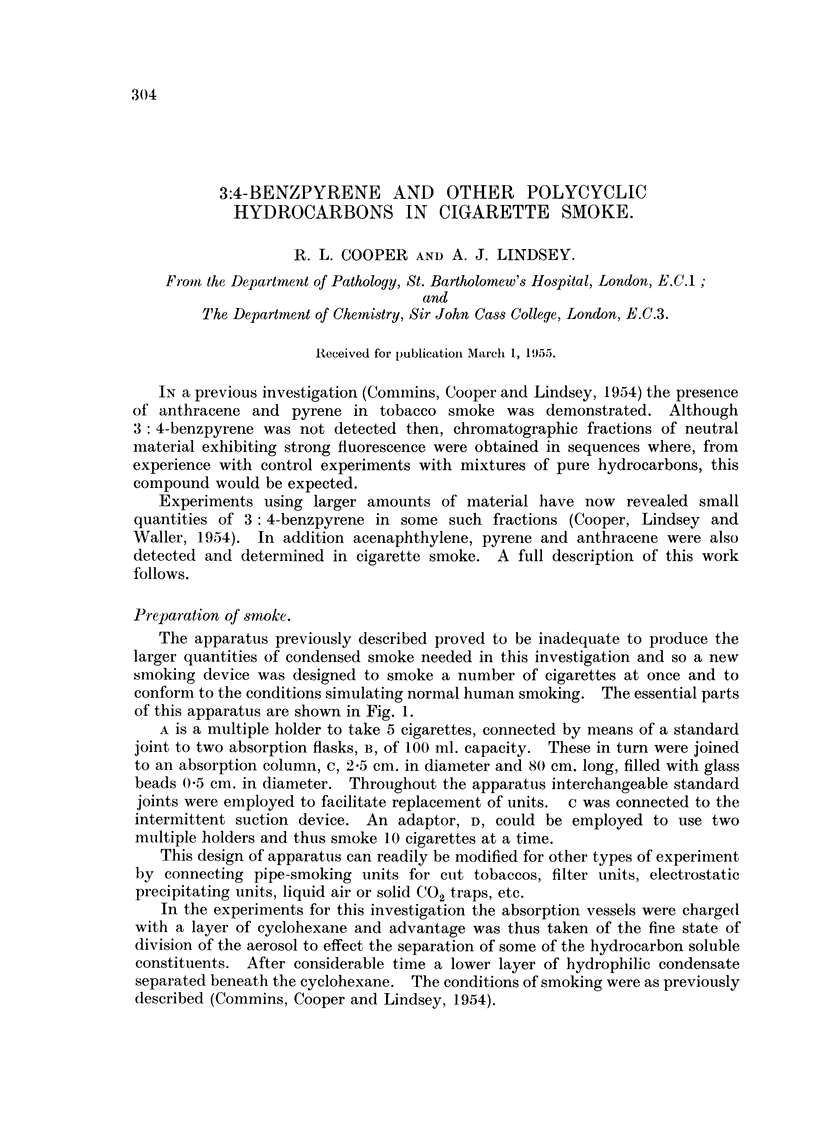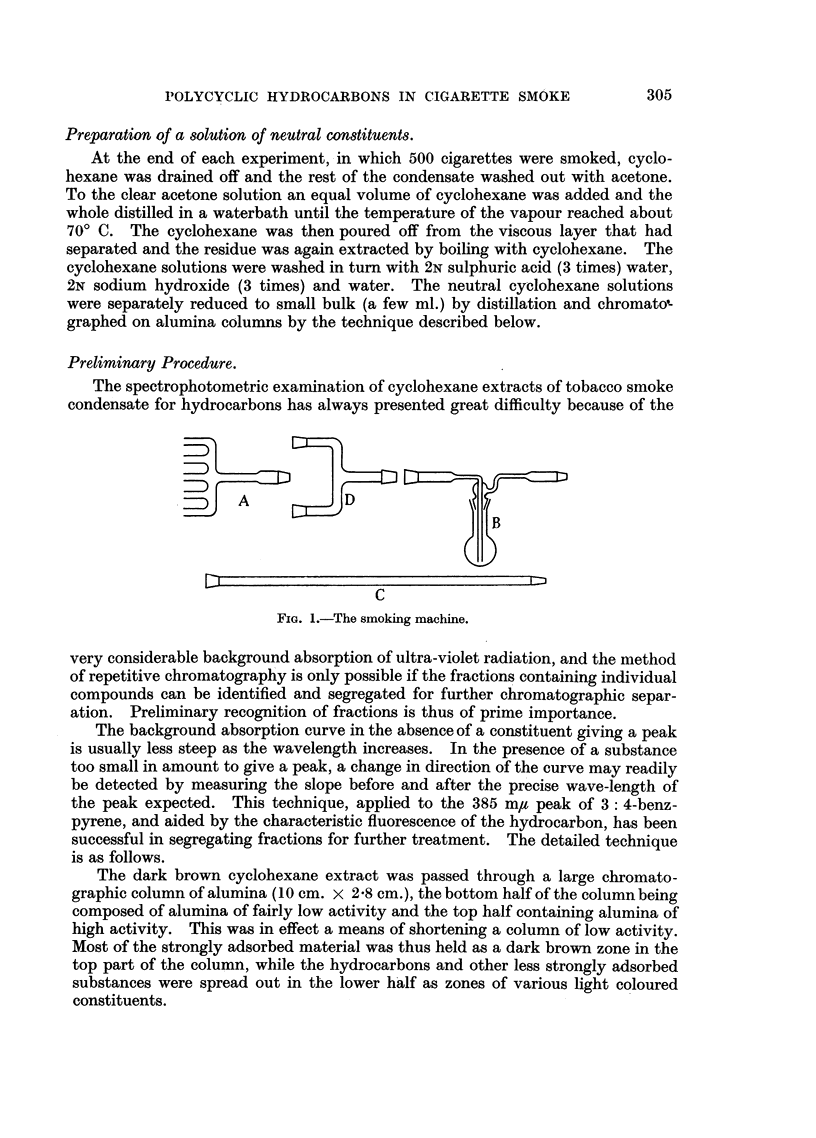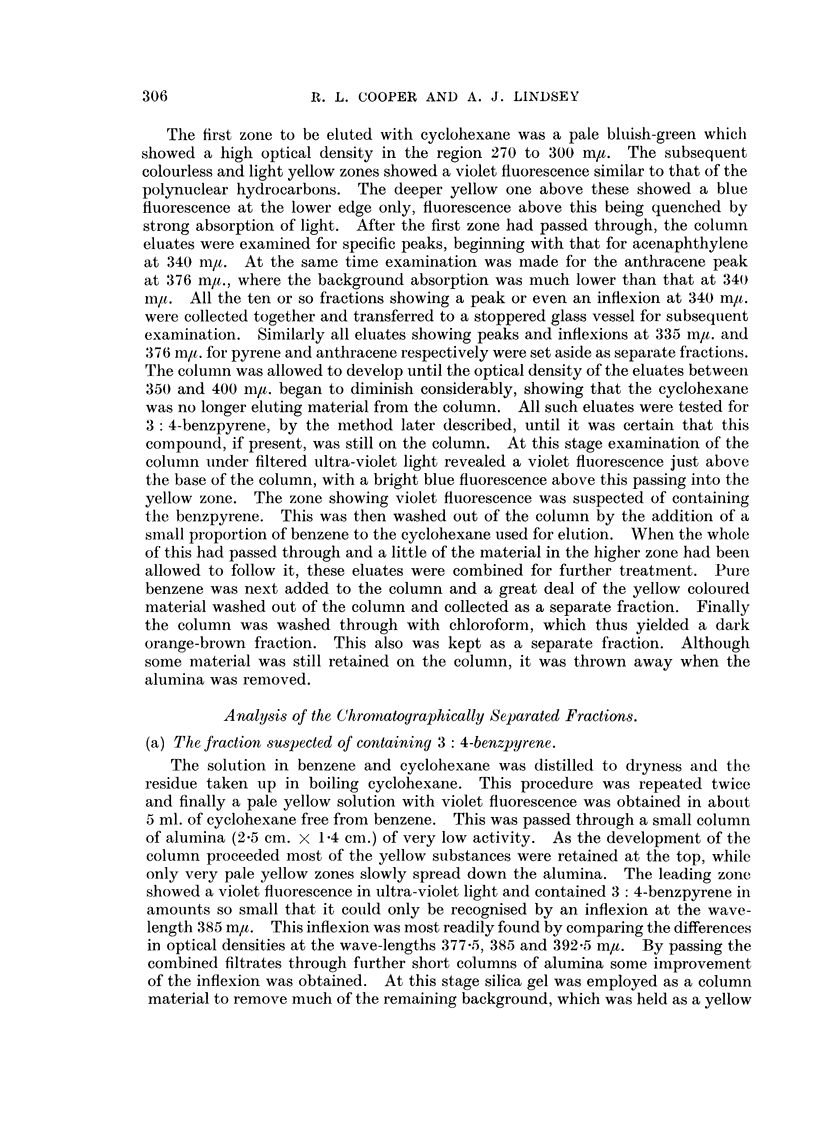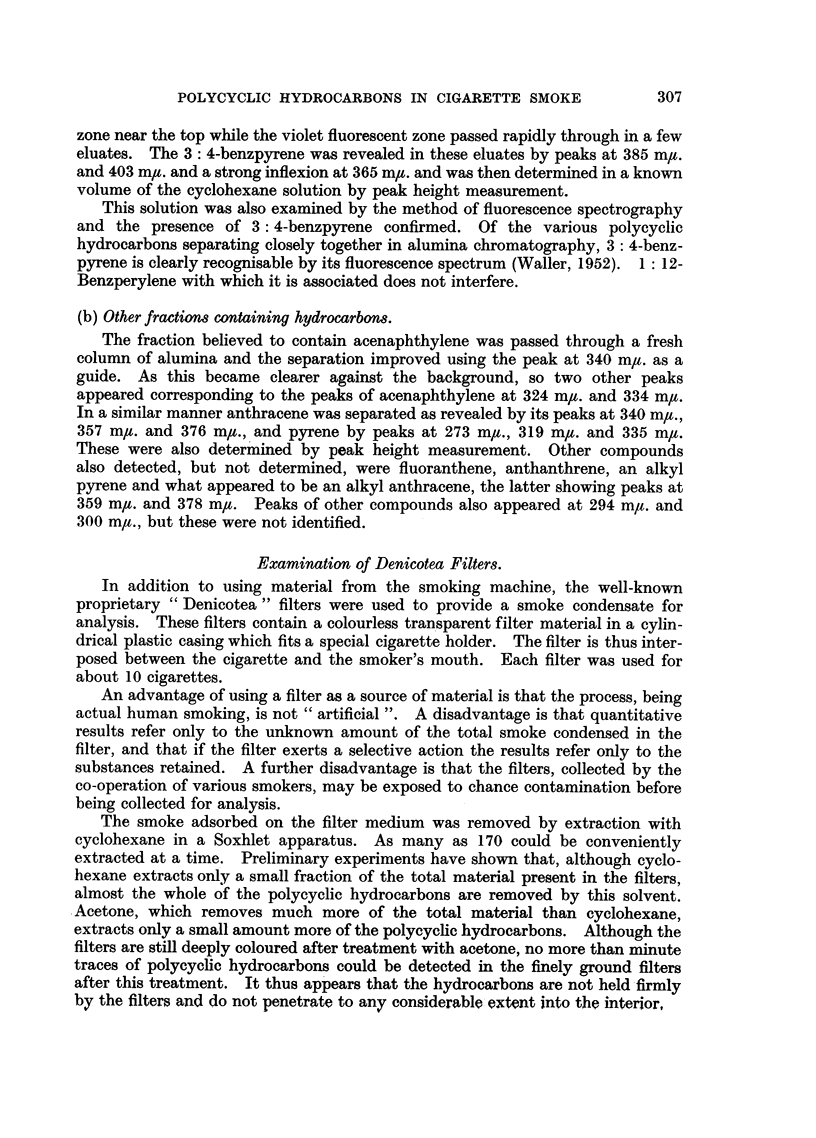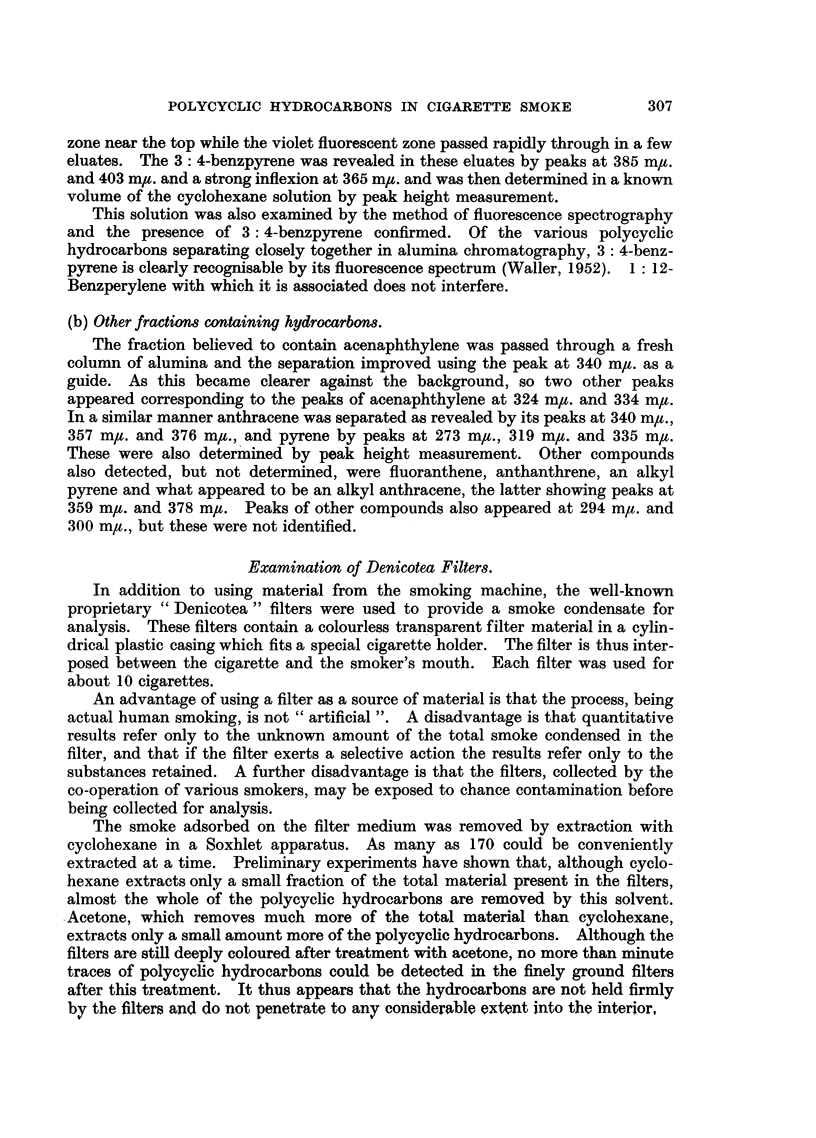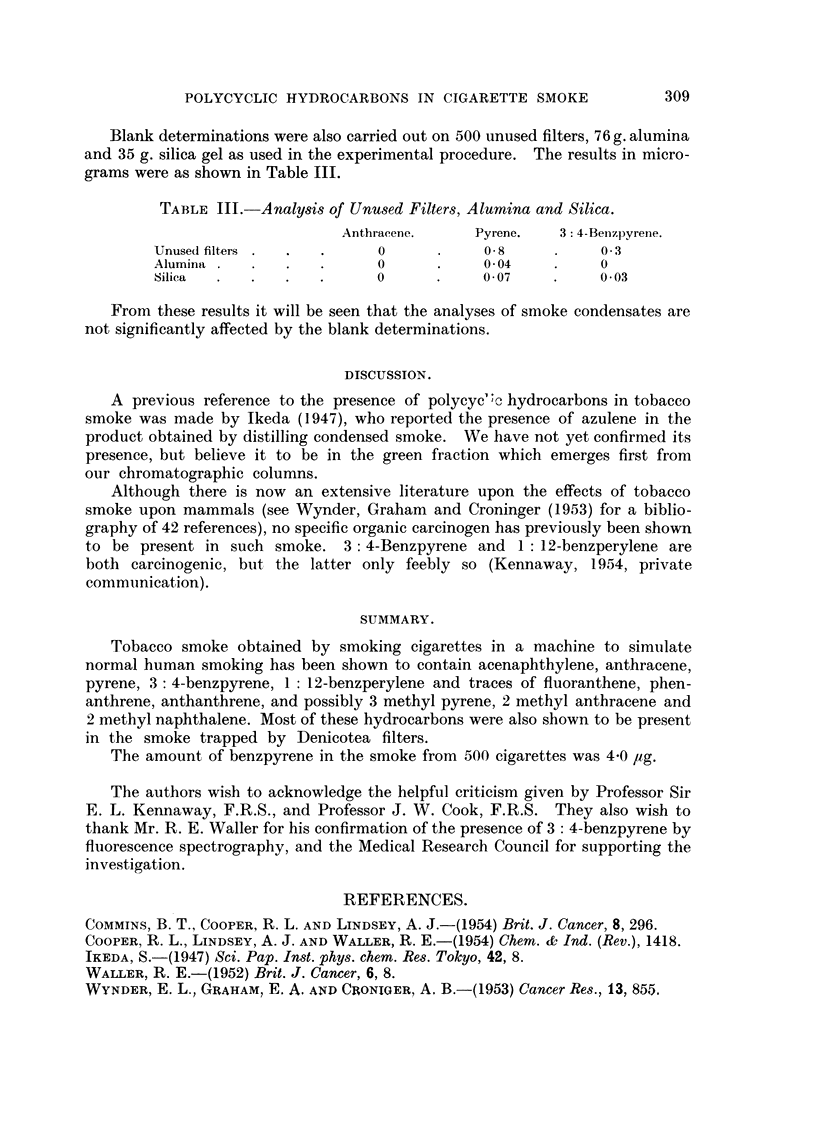# 3:4-Benzpyrene and Other Polycyclic Hydrocarbons in Cigarette Smoke

**DOI:** 10.1038/bjc.1955.27

**Published:** 1955-06

**Authors:** R. L. Cooper, A. J. Lindsey


### 3:4-BENZPYRENE AND OTHER POLYCYCLIC HYDROCARBONS IN CIGARETTE SMOKE.

R. L. COOPER AND A. J. LINDSEY.

*From the Department of Pathology, St. Bartholomew's Hospital, London, E.C.1 ;*
*and*
*The Department of Chemistry, Sir John Cass College, London, E.C.3.*

Received for publication March 1, 1955.

IN a previous investigation (Commins, Cooper and Lindsey, 1954) the presence of anthracene and pyrene in tobacco smoke was demonstrated. Although 3 : 4-benzpyrene was not detected then, chromatographic fractions of neutral material exhibiting strong fluorescence were obtained in sequences where, from experience with control experiments with mixtures of pure hydrocarbons, this compound would be expected.

Experiments using larger amounts of material have now revealed small quantities of 3 : 4-benzpyrene in some such fractions (Cooper, Lindsey and Waller, 1954). In addition acenaphthylene, pyrene and anthracene were also detected and determined in cigarette smoke. A full description of this work follows.

*Preparation of smoke.*

The apparatus previously described proved to be inadequate to produce the larger quantities of condensed smoke needed in this investigation and so a new smoking device was designed to smoke a number of cigarettes at once and to conform to the conditions simulating normal human smoking. The essential parts of this apparatus are shown in Fig. 1.

A is a multiple holder to take 5 cigarettes, connected by means of a standard joint to two absorption flasks, B, of 100 ml. capacity. These in turn were joined to an absorption column, C, 2·5 cm. in diameter and 80 cm. long, filled with glass beads 0·5 cm. in diameter. Throughout the apparatus interchangeable standard joints were employed to facilitate replacement of units. C was connected to the intermittent suction device. An adaptor, D, could be employed to use two multiple holders and thus smoke 10 cigarettes at a time.

This design of apparatus can readily be modified for other types of experiment by connecting pipe-smoking units for cut tobaccos, filter units, electrostatic precipitating units, liquid air or solid $CO_2$ traps, etc.

In the experiments for this investigation the absorption vessels were charged with a layer of cyclohexane and advantage was thus taken of the fine state of division of the aerosol to effect the separation of some of the hydrocarbon soluble constituents. After considerable time a lower layer of hydrophilic condensate separated beneath the cyclohexane. The conditions of smoking were as previously described (Commins, Cooper and Lindsey, 1954).

*Preparation of a solution of neutral constituents.*

At the end of each experiment, in which 500 cigarettes were smoked, cyclohexane was drained off and the rest of the condensate washed out with acetone. To the clear acetone solution an equal volume of cyclohexane was added and the whole distilled in a waterbath until the temperature of the vapour reached about 70° C. The cyclohexane was then poured off from the viscous layer that had separated and the residue was again extracted by boiling with cyclohexane. The cyclohexane solutions were washed in turn with 2N sulphuric acid (3 times) water, 2N sodium hydroxide (3 times) and water. The neutral cyclohexane solutions were separately reduced to small bulk (a few ml.) by distillation and chromatographed on alumina columns by the technique described below.

*Preliminary Procedure.*

The spectrophotometric examination of cyclohexane extracts of tobacco smoke condensate for hydrocarbons has always presented great difficulty because of the

FIG. 1.—The smoking machine.

very considerable background absorption of ultra-violet radiation, and the method of repetitive chromatography is only possible if the fractions containing individual compounds can be identified and segregated for further chromatographic separation. Preliminary recognition of fractions is thus of prime importance.

The background absorption curve in the absence of a constituent giving a peak is usually less steep as the wavelength increases. In the presence of a substance too small in amount to give a peak, a change in direction of the curve may readily be detected by measuring the slope before and after the precise wave-length of the peak expected. This technique, applied to the 385 m$\mu$ peak of 3 : 4-benzpyrene, and aided by the characteristic fluorescence of the hydrocarbon, has been successful in segregating fractions for further treatment. The detailed technique is as follows.

The dark brown cyclohexane extract was passed through a large chromatographic column of alumina (10 cm. × 2·8 cm.), the bottom half of the column being composed of alumina of fairly low activity and the top half containing alumina of high activity. This was in effect a means of shortening a column of low activity. Most of the strongly adsorbed material was thus held as a dark brown zone in the top part of the column, while the hydrocarbons and other less strongly adsorbed substances were spread out in the lower half as zones of various light coloured constituents.

The first zone to be eluted with cyclohexane was a pale bluish-green which showed a high optical density in the region 270 to 300 m$\mu$. The subsequent colourless and light yellow zones showed a violet fluorescence similar to that of the polynuclear hydrocarbons. The deeper yellow one above these showed a blue fluorescence at the lower edge only, fluorescence above this being quenched by strong absorption of light. After the first zone had passed through, the column eluates were examined for specific peaks, beginning with that for acenaphthylene at 340 m$\mu$. At the same time examination was made for the anthracene peak at 376 m$\mu$., where the background absorption was much lower than that at 340 m$\mu$. All the ten or so fractions showing a peak or even an inflexion at 340 m$\mu$. were collected together and transferred to a stoppered glass vessel for subsequent examination. Similarly all eluates showing peaks and inflexions at 335 m$\mu$. and 376 m$\mu$. for pyrene and anthracene respectively were set aside as separate fractions. The column was allowed to develop until the optical density of the eluates between 350 and 400 m$\mu$. began to diminish considerably, showing that the cyclohexane was no longer eluting material from the column. All such eluates were tested for 3 : 4-benzpyrene, by the method later described, until it was certain that this compound, if present, was still on the column. At this stage examination of the column under filtered ultra-violet light revealed a violet fluorescence just above the base of the column, with a bright blue fluorescence above this passing into the yellow zone. The zone showing violet fluorescence was suspected of containing the benzpyrene. This was then washed out of the column by the addition of a small proportion of benzene to the cyclohexane used for elution. When the whole of this had passed through and a little of the material in the higher zone had been allowed to follow it, these eluates were combined for further treatment. Pure benzene was next added to the column and a great deal of the yellow coloured material washed out of the column and collected as a separate fraction. Finally the column was washed through with chloroform, which thus yielded a dark orange-brown fraction. This also was kept as a separate fraction. Although some material was still retained on the column, it was thrown away when the alumina was removed.

### *Analysis of the Chromatographically Separated Fractions.*

(a) *The fraction suspected of containing* 3 : 4-*benzpyrene.*

The solution in benzene and cyclohexane was distilled to dryness and the residue taken up in boiling cyclohexane. This procedure was repeated twice and finally a pale yellow solution with violet fluorescence was obtained in about 5 ml. of cyclohexane free from benzene. This was passed through a small column of alumina (2·5 cm. $\times$ 1·4 cm.) of very low activity. As the development of the column proceeded most of the yellow substances were retained at the top, while only very pale yellow zones slowly spread down the alumina. The leading zone showed a violet fluorescence in ultra-violet light and contained 3 : 4-benzpyrene in amounts so small that it could only be recognised by an inflexion at the wave-length 385 m$\mu$. This inflexion was most readily found by comparing the differences in optical densities at the wave-lengths 377·5, 385 and 392·5 m$\mu$. By passing the combined filtrates through further short columns of alumina some improvement of the inflexion was obtained. At this stage silica gel was employed as a column material to remove much of the remaining background, which was held as a yellow zone near the top while the violet fluorescent zone passed rapidly through in a few eluates. The 3 : 4-benzpyrene was revealed in these eluates by peaks at 385 m$\mu$. and 403 m$\mu$. and a strong inflexion at 365 m$\mu$. and was then determined in a known volume of the cyclohexane solution by peak height measurement.

This solution was also examined by the method of fluorescence spectrography and the presence of 3 : 4-benzpyrene confirmed. Of the various polycyclic hydrocarbons separating closely together in alumina chromatography, 3 : 4-benzpyrene is clearly recognisable by its fluorescence spectrum (Waller, 1952). 1 : 12-Benzperylene with which it is associated does not interfere.

(b) *Other fractions containing hydrocarbons.*

The fraction believed to contain acenaphthylene was passed through a fresh column of alumina and the separation improved using the peak at 340 m$\mu$. as a guide. As this became clearer against the background, so two other peaks appeared corresponding to the peaks of acenaphthylene at 324 m$\mu$. and 334 m$\mu$. In a similar manner anthracene was separated as revealed by its peaks at 340 m$\mu$., 357 m$\mu$. and 376 m$\mu$., and pyrene by peaks at 273 m$\mu$., 319 m$\mu$. and 335 m$\mu$. These were also determined by peak height measurement. Other compounds also detected, but not determined, were fluoranthene, anthanthrene, an alkyl pyrene and what appeared to be an alkyl anthracene, the latter showing peaks at 359 m$\mu$. and 378 m$\mu$. Peaks of other compounds also appeared at 294 m$\mu$. and 300 m$\mu$., but these were not identified.

### *Examination of Denicotea Filters.*

In addition to using material from the smoking machine, the well-known proprietary " Denicotea " filters were used to provide a smoke condensate for analysis. These filters contain a colourless transparent filter material in a cylindrical plastic casing which fits a special cigarette holder. The filter is thus interposed between the cigarette and the smoker's mouth. Each filter was used for about 10 cigarettes.

An advantage of using a filter as a source of material is that the process, being actual human smoking, is not " artificial ". A disadvantage is that quantitative results refer only to the unknown amount of the total smoke condensed in the filter, and that if the filter exerts a selective action the results refer only to the substances retained. A further disadvantage is that the filters, collected by the co-operation of various smokers, may be exposed to chance contamination before being collected for analysis.

The smoke adsorbed on the filter medium was removed by extraction with cyclohexane in a Soxhlet apparatus. As many as 170 could be conveniently extracted at a time. Preliminary experiments have shown that, although cyclohexane extracts only a small fraction of the total material present in the filters, almost the whole of the polycyclic hydrocarbons are removed by this solvent. Acetone, which removes much more of the total material than cyclohexane, extracts only a small amount more of the polycyclic hydrocarbons. Although the filters are still deeply coloured after treatment with acetone, no more than minute traces of polycyclic hydrocarbons could be detected in the finely ground filters after this treatment. It thus appears that the hydrocarbons are not held firmly by the filters and do not penetrate to any considerable extent into the interior.

zone near the top while the violet fluorescent zone passed rapidly through in a few eluates. The 3 : 4-benzpyrene was revealed in these eluates by peaks at 385 mμ. and 403 mμ. and a strong inflexion at 365 mμ. and was then determined in a known volume of the cyclohexane solution by peak height measurement.

This solution was also examined by the method of fluorescence spectrography and the presence of 3 : 4-benzpyrene confirmed. Of the various polycyclic hydrocarbons separating closely together in alumina chromatography, 3 : 4-benzpyrene is clearly recognisable by its fluorescence spectrum (Waller, 1952). 1 : 12-Benzperylene with which it is associated does not interfere.

(b) *Other fractions containing hydrocarbons.*

The fraction believed to contain acenaphthylene was passed through a fresh column of alumina and the separation improved using the peak at 340 mμ. as a guide. As this became clearer against the background, so two other peaks appeared corresponding to the peaks of acenaphthylene at 324 mμ. and 334 mμ. In a similar manner anthracene was separated as revealed by its peaks at 340 mμ., 357 mμ. and 376 mμ., and pyrene by peaks at 273 mμ., 319 mμ. and 335 mμ. These were also determined by peak height measurement. Other compounds also detected, but not determined, were fluoranthene, anthanthrene, an alkyl pyrene and what appeared to be an alkyl anthracene, the latter showing peaks at 359 mμ. and 378 mμ. Peaks of other compounds also appeared at 294 mμ. and 300 mμ., but these were not identified.

### *Examination of Denicotea Filters.*

In addition to using material from the smoking machine, the well-known proprietary " Denicotea " filters were used to provide a smoke condensate for analysis. These filters contain a colourless transparent filter material in a cylindrical plastic casing which fits a special cigarette holder. The filter is thus interposed between the cigarette and the smoker's mouth. Each filter was used for about 10 cigarettes.

An advantage of using a filter as a source of material is that the process, being actual human smoking, is not " artificial ". A disadvantage is that quantitative results refer only to the unknown amount of the total smoke condensed in the filter, and that if the filter exerts a selective action the results refer only to the substances retained. A further disadvantage is that the filters, collected by the co-operation of various smokers, may be exposed to chance contamination before being collected for analysis.

The smoke adsorbed on the filter medium was removed by extraction with cyclohexane in a Soxhlet apparatus. As many as 170 could be conveniently extracted at a time. Preliminary experiments have shown that, although cyclohexane extracts only a small fraction of the total material present in the filters, almost the whole of the polycyclic hydrocarbons are removed by this solvent. Acetone, which removes much more of the total material than cyclohexane, extracts only a small amount more of the polycyclic hydrocarbons. Although the filters are still deeply coloured after treatment with acetone, no more than minute traces of polycyclic hydrocarbons could be detected in the finely ground filters after this treatment. It thus appears that the hydrocarbons are not held firmly by the filters and do not penetrate to any considerable extent into the interior,

Blank determinations were also carried out on 500 unused filters, 76 g. alumina and 35 g. silica gel as used in the experimental procedure. The results in micrograms were as shown in Table III.

TABLE III.—*Analysis of Unused Filters, Alumina and Silica.*

| | Anthracene. | Pyrene. | 3 : 4-Benzpyrene. |
|---|---|---|---|
| Unused filters | 0 | 0·8 | 0·3 |
| Alumina | 0 | 0·04 | 0 |
| Silica | 0 | 0·07 | 0·03 |

From these results it will be seen that the analyses of smoke condensates are not significantly affected by the blank determinations.

### DISCUSSION.

A previous reference to the presence of polycyc'ic hydrocarbons in tobacco smoke was made by Ikeda (1947), who reported the presence of azulene in the product obtained by distilling condensed smoke. We have not yet confirmed its presence, but believe it to be in the green fraction which emerges first from our chromatographic columns.

Although there is now an extensive literature upon the effects of tobacco smoke upon mammals (see Wynder, Graham and Croninger (1953) for a bibliography of 42 references), no specific organic carcinogen has previously been shown to be present in such smoke. 3 : 4-Benzpyrene and 1 : 12-benzperylene are both carcinogenic, but the latter only feebly so (Kennaway, 1954, private communication).

### SUMMARY.

Tobacco smoke obtained by smoking cigarettes in a machine to simulate normal human smoking has been shown to contain acenaphthylene, anthracene, pyrene, 3 : 4-benzpyrene, 1 : 12-benzperylene and traces of fluoranthene, phenanthrene, anthanthrene, and possibly 3 methyl pyrene, 2 methyl anthracene and 2 methyl naphthalene. Most of these hydrocarbons were also shown to be present in the smoke trapped by Denicotea filters.

The amount of benzpyrene in the smoke from 500 cigarettes was 4·0 $\mu$g.

The authors wish to acknowledge the helpful criticism given by Professor Sir E. L. Kennaway, F.R.S., and Professor J. W. Cook, F.R.S. They also wish to thank Mr. R. E. Waller for his confirmation of the presence of 3 : 4-benzpyrene by fluorescence spectrography, and the Medical Research Council for supporting the investigation.

### REFERENCES.

COMMINS, B. T., COOPER, R. L. AND LINDSEY, A. J.—(1954) *Brit. J. Cancer*, **8**, 296.
COOPER, R. L., LINDSEY, A. J. AND WALLER, R. E.—(1954) *Chem. & Ind.* (*Rev.*), 1418.
IKEDA, S.—(1947) *Sci. Pap. Inst. phys. chem. Res. Tokyo*, **42**, 8.
WALLER, R. E.—(1952) *Brit. J. Cancer*, **6**, 8.
WYNDER, E. L., GRAHAM, E. A. AND CRONIGER, A. B.—(1953) *Cancer Res.*, **13**, 855.